# Prediction of atrial fibrillation admissions in arrhythmia naïve patients from structured electronic health record data

**DOI:** 10.1186/s12911-025-03199-x

**Published:** 2025-09-29

**Authors:** Tanmay Gokhale, Nirav R. Bhatt, Matthew Starr, Suresh Mulukutla, Floyd Thoma, Murat Akcakaya, Salah Al-Zaiti, Raul G. Nogueira, Samir Saba

**Affiliations:** 1 Heart and Vascular Institute, University of Pittsburgh Medical Center, 200 Lothrop Street, South Tower, Pittsburgh, PA E355.6 USA; 2 Neurology Department, University of Pittsburgh, Pittsburgh, Pennsylvania USA; 3 Swanson School of Engineering at the University of Pittsburgh, Pittsburgh, Pennsylvania USA; 4 Nursing School at the University of Rochester, Rochester, New York USA

**Keywords:** Atrial fibrillation, Electronic health records, Prediction, Admission, Machine learning

## Abstract

**Background:**

Atrial fibrillation (AF) is the most prevalent sustained arrhythmia, but its diagnosis is often elusive. In this study, we examined the role of machine learning (ML) algorithms in predicting AF in arrhythmia-naïve patients, based on structured domains of the electronic health records (EHR).

**Methods:**

Patients (*N* = 186,769) with no prior history of AF, who received at least 1 echocardiogram and who had a minimum of 3 months of follow-up, were included. Data from the EHR were grouped into domains (demographic; social determinants of health; past medical history, medications, electrocardiogram (EKG), and echocardiogram (Echo)) and tested incrementally for their ability to predict incident AF admission to the hospital.

**Results:**

Of the overall cohort, 4,751 (2.5%) patients were admitted for AF over a median follow-up time of 35 months. Incremental EHR domains increased the area under the receiver-operator curve (AUROC) for all ML classifiers, with Gradient Boosting achieving an AUROC of 0.85 when all domains were included, but with a poor F1 score of 14% at the maximal Youden index. Using the EKG and Echo domains alone achieved comparable performance to when all EHR domains were included. These results were externally validated.

**Conclusion:**

More domains of structured EHR improve the ability to predict incident AF admissions but structured EKG and Echo domains realize the most gain. Although ML models exhibited good discrimination, the precision is poor due to the low event rate.

**Supplementary information:**

The online version contains supplementary material available at 10.1186/s12911-025-03199-x.

## Background

Atrial fibrillation (AF) is the most common sustained arrhythmia [1–4] and is associated with significant morbidity such as heart failure [5, 6] and thromboembolic events, including strokes [7], as well as mortality [8–10]. AF can be elusive to diagnosis given its often paroxysmal and asymptomatic nature [4] but still accounts for about 15% of all ischemic strokes, even when patients do not have a diagnosis of AF at the time of the cerebrovascular event. Because of these considerations, predicting the risk of developing AF before it happens is extremely important to provide patients with adequate protection, as early as possible, and prevent adverse clinical outcomes.

Developing new tools to predict the future occurrence of AF is therefore of utmost importance with immediate clinical implications to patient care. In the present study, we examined the performance of several machine learning (ML) classifiers applied to data from structured domains in the electronic health records (EHR) of a large, multi-site healthcare delivery system, for predicting the incidence of AF hospital admissions, in patients with no prior history of AF.

## Methods

### Study design and dataset

This study was conducted in accordance with the ethical principles of the Declaration of Helsinki and the Belmont report and was approved by the University of Pittsburgh internal review board, who waived the need for informed consent, given the observational nature of the study. No funding was available for this research.

**Fig. 1:**
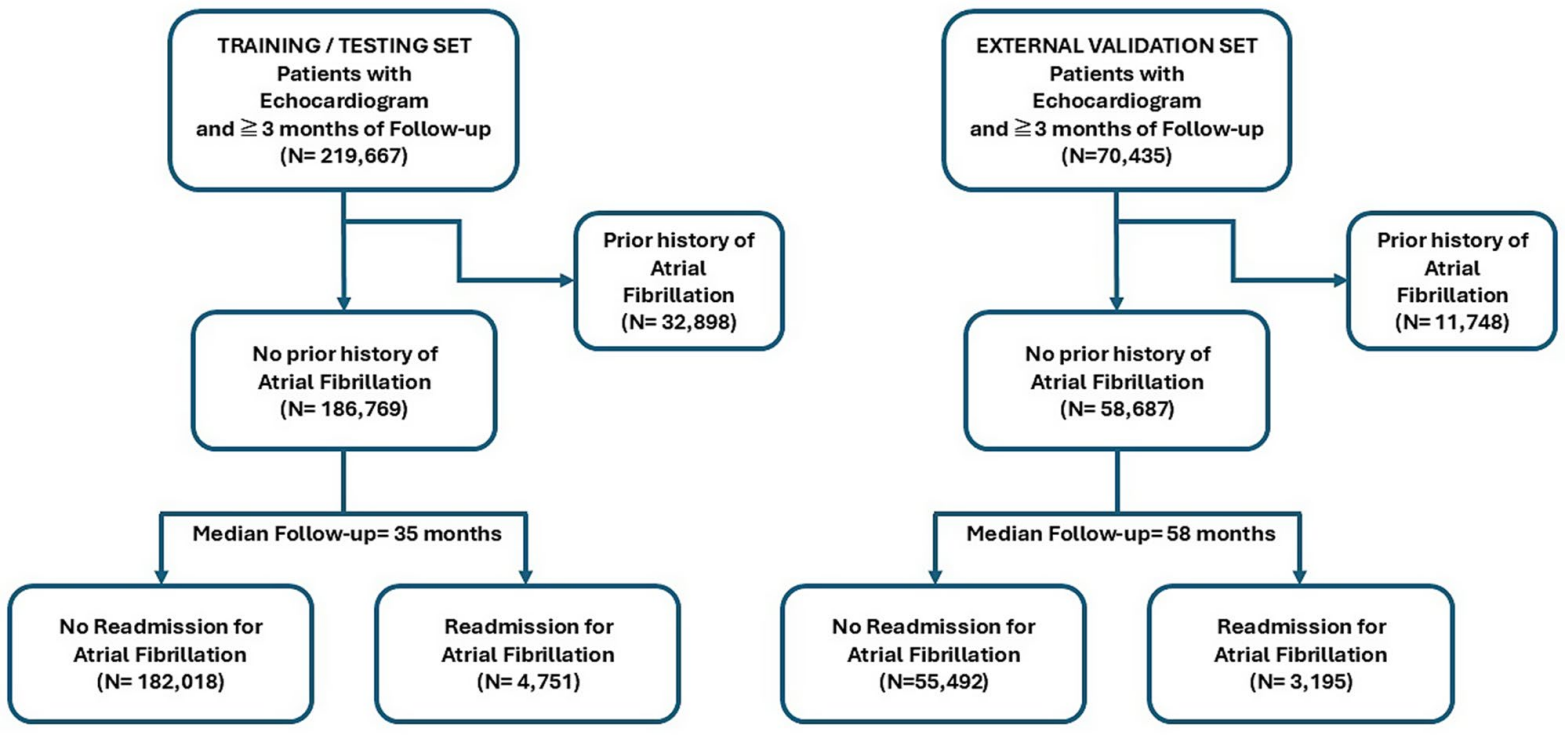
Consort diagram detailing the overall cohort of patients included in the training and testing (left panel) as well as in the external validation (right panel) analyses, stratified by incident hospitalization for atrial fibrillation over follow-up

Data were collected from the University of Pittsburgh analytic data warehouse. The data underlying this article will be shared upon reasonable request to the corresponding author. Consecutive patients who underwent transthoracic echocardiographic (Echo) examination at our institution between 2010-2022 and who had ≥3 months follow-up from the time of the Echo were included. Patients with prior history of AF were excluded. Patients were followed up to the primary endpoint of first admission to the hospital with a primary diagnosis of paroxysmal, persistent, or unspecified AF as indicated by *International Classification of Diseases* codes (427.31, 427.32, I48.-). A CONSORT diagram detailing the patient cohort is shown in Fig. 1. Briefly, of the initial cohort of 219,667 patients who underwent Echo testing and had a minimum of 3 months follow-up at our institution 32,898 (15%) were excluded due to a prior diagnosis of AF. Of the remaining cohort of 186,769 patients, 4,751 (2.5%) patients were admitted to the hospital with a primary diagnosis of AF over a median follow-up of 35 months. Model performance was externally validated using a cohort of 58,687 patients without prior history of AF, with an AF admission event rate of 3,195 (5.8%) over a median follow-up time of 58 months, recruited from the University of Pittsburgh Pinnacle hospital in Harrisburg, PA which was not part of the training or testing cohort (Fig. 1). Unfortunately, 5 variables (3 EKG variables – the axes of the P, R and T waves and 2 Echo variables – the presence and grade of diastolic dysfunction) could not be obtained in the validation dataset. In addition, the follow-up time was also significantly longer in the validation dataset (median = 58 months) compared to the derivation dataset (median = 35 months). To overcome these significant differences, we reran our model on both the derivation and external validation datasets, after excluding the 5 missing variables from both datasets and with setting a fixed time horizon ( = 35 months) to assess model discrimination.

### Statistical analyses

Continuous data are presented as mean ± standard deviation and were compared between patients who were admitted for AF and those who were not using the t test. Categorical data are reported as frequencies with percentages and were compared using Chi-square test. Data from the EHR were grouped into the following domains, as detailed in Table 1: Demographic data, social determinants of health, past medical history, medications, structured electrocardiographic (EKG) and Echo parameters. Missing data were imputed to the mean of continuous variables and to the mode of categorical variables. ML models were developed using 5 classifiers (Logistic Regression, Decision Tree, Random Forest, Gradient Boosting and Neural Network) to calculate the accuracy, area under the receiver-operator curve (AUROC), sensitivity, specificity, positive (PPV) and negative (NPV) predictive values, and the F1 score, which is the harmonic of precision and recall [11, 12] (F1 = 2 ×sensitivity ×PPV/(sensitivity + PPV)). These parameters were reported at the threshold that maximizes Youden’s index, which is equal to sensitivity + specificity − 1 [13]. Models were trained on 80% of the dataset and tested on the remaining 20%. In all analyses, the incremental gain from added EHR domains was statistically tested using linear regression analysis, Spearman’s rank correlation, and Kendall’s Tau test. Further analyses were performed to examine if a reduced number of EHR domains would achieve comparable performance to the full set of domains. In addition, we examined the performance of the ML models as a survival analysis using the Random Survival Forest method, to examine if survival analyses lead to better model performance. Performance of the survival models was evaluated at 3 time-horizons: 12, 24, and 36 months. Statistical significance was tested at the 0.05 p value cutoff. All analyses were performed using open-source Python programming (Python Software Foundation, Wilmington, Delaware).

**Table 1:** Baseline characteristics divided by domains of the electronic health record and stratified by atrial fibrillation status

	No AF Admission (N = 182,018)	AF Admission (N = 4,751)	P value
**Demographics**			
Age (years)	60 ± 18	71 ± 13	< 0.001
Women	101,013 (55.5%)	2,304 (48.5%)	< 0.001
Race (White)	159,310 (87.5%))	4,341 (91.4%)	< 0.001
Height (cm)	169 ± 11	169 ± 12	< 0.001
Weight (kgs)	86 ± 24	87 ± 23	< 0.001
Body Mass Index (kg/m^2^)	30 ± 8	30 ± 7	0.32
**Social Determinants of Health**			
Zip Code of Residence	–	–	< 0.001
Estimated Median Household Income (USD)	52,821 ± 18,681	53,219 ± 18,887	0.15
Current Tobacco Use	30,304 (16.6%)	633 (13.3%)	< 0.001
**Past Medical History**			
Cancer	29,645 (16.3%)	959 (20.2%)	< 0.001
Lung Cancer	2,121 (1.2%)	88 (1.9%)	< 0.001
Obesity	98,808 (54.3%)	2,669 (56.2%)	0.010
Morbid Obesity	28,020 (15.4%)	684 (14.4%)	0.06
Chronic Obstructive Pulmonary Disease	19,845 (10.9%)	719 (15.1%)	< 0.001
Asthma	48,907 (26.9%)	1,287 (27.1%)	0.74
Pulmonary Hypertension	5,309 (2.9%)	193 (4.1%)	< 0.001
Obstructive Sleep Apnea	25,374 (13.9%)	685 (14.4%)	0.35
Stroke	14,919 (8.2%)	473 (10%)	< 0.001
Transient Ischemic Attack	5,213 (2.9%)	204 (4.3%)	< 0.001
Hemorrhagic Stroke	1,139 (0.6%)	29 (0.6%)	0.99
Major Bleeding	29,574 (16.2%)	840 (17.7%)	0.009
Hypertension	92,250 (50.7%	2,939 (61.9%)	< 0.001
Diabetes Mellitus	36,123 (19.8%)	1,100 (23.2%)	< 0.001
Hyperlipidemia	86,760 (47.7%)	2,629 (55.3%)	< 0.001
Coronary Artery Disease	34,573 (19.0%)	1,371 (28.9%)	< 0.001
Hypertrophic Cardiomyopathy	646 (0.4%)	29 (0.6%)	0.022
Congestive Heart Failure	13,539 (7.4%)	496 (10.4%)	< 0.001
Cardiac Arrest	354 (0.2%)	8 (0.2%)	0.87
Ventricular Tachycardia or Fibrillation	173 (0.1%)	8 (0.2%)	0.15
Arterial Vascular Disease	11,697 (6.4%)	416 (8.8%)	< 0.001
Deep Vein Thrombosis	5,252 (2.9%)	143 (3.0%)	0.60
Pulmonary Embolism	4,939 (2.7%)	155 (3.3%)	0.022
Antiphospholipid Antibody	205 (0.1%)	2 (0.0%)	0.18
Acute Limb Ischemia	696 (0.4%)	36 (0.8%)	< 0.001
Ischemic Bowels	164 (0.1%)	8 (0.2%)	0.09
Chronic Kidney Disease	13,155 (7.2%)	120 (8.8%)	< 0.001
End-Stage Renal Disease	3,055 (1.7%)	95 (2.0%)	0.95
Severe Liver Disease	816 (0.4%)	15 (0.3%)	0.23
Amyloidosis	168 (0.1%)	2 (0.0%)	0.25
Sarcoidosis	1,035 (0.6%)	29 (0.6%)	0.91
CHA_2_DS2-Vasc score	2.2 ± 1.6	2.8 ± 1.6	< 0.001
Charlson comorbidity index	1.18 ± 1.56	1.41 ± 1.57	< 0.001
Elixhauser van Walraven score	3.77 ± 6.58	4.59 ± 6.64	< 0.001
**Medications**			
Angiotensin Converting Enzyme inhibitor	44,958 (25.0%)	1,508 (31.8%)	< 0.001
Angiotensin Receptor Blocker	27,238 (15.2%)	915 (19.3%)	< 0.001
Angiotensin Receptor Neprilysin Inhibitor	1,255 (0.7%)	24 (0.5%)	0.14
Beta Blocker	62,514 (34.8%)	2,471 (52.0%)	< 0.001
Mineralocorticoid Receptor Antagonist	10,920 (6.1%)	261 (5.5%)	0.10
Sodium Glucose Cotransporter inhibitor	2,931 (1.6%)	41 (0.9%)	< 0.001
Aspirin	67,762 (37.7%)	2,520 (53.1%)	< 0.001
Oral Anticoagulation	11,989 (7.5%)	769 (16.5%)	< 0.001
**Electrcardiogram**			
Heart Rate (b/min)	80 ± 21	85 ± 28	< 0.001
PR Interval (ms)	161 ± 32	171 ± 37	< 0.001
QRS Width (ms)	43 ± 34	43 ± 36	0.87
QT Interval (ms)	393 ± 47	391 ± 56	0.013
QTc Interval (ms)	445 ± 36	450 ± 39	< 0.001
P Axis (degrees)	50 ± 24	54 ± 35	< 0.001
R Axis (degrees)	45 ± 39	45 ± 43	0.85
T Axis (degrees)	50 ± 34	55 ± 39	< 0.001
**Echocardiogram**			
Number of Echocardiograms	2.21 ± 1.97	3.94 ± 2.90	< 0.001
Left Ventricular Ejection Fraction (%)	56 ± 9	54 ± 11	< 0.001
Lowest LVEF is Normal	156,080 (85.7%)	3,369 (70.9%)	< 0.001
Highest LVEF is Normal	171,228 (94.1%)	4,378 (92.1%)	< 0.001
Left Ventricular End-Systolic Diameter (cm)	3.1 ± 1.6	3.2 ± 0.8	< 0.001
Left Ventricular End-Diastolic Diameter (cm)	4.5 ± 0.7	4.6 ± 0.8	< 0.001
Left Ventricular Mass (grams)	189 ± 4493	198 ± 71	0.90
Left Ventricular Mass Index (grams/m^2^)	99 ± 3467	98 ± 32	0.99
Left Posterior Wall Thickness (cm)	1.0 ± 0.2	1.1 ± 0.2	< 0.001
Intraventricular Septal Thickness (cm)	1.1 ± 0.4	1.2 ± 0.2	< 0.001
Left Atrial Diameter (cm)	3.7 ± 1.8	4.0 ± 0.7	< 0.001
**Diastolic Dysfunction Grade**			
*No Diastolic Dysfunction*	110,725 (60.8%)	1,928 (40.6%)	< 0.001
*Grade I*	21,863 (12.0%)	752 (15.8%)	
*Grade II*	6,423 (3.5%)	372 (7.8%)	
*Grade III*	1,184 (0.7%)	82 (1.7%)	
*Cannot be determined*	41,823 (23.0%)	1,617 (34.0%)	

## Results

**Fig. 2:**
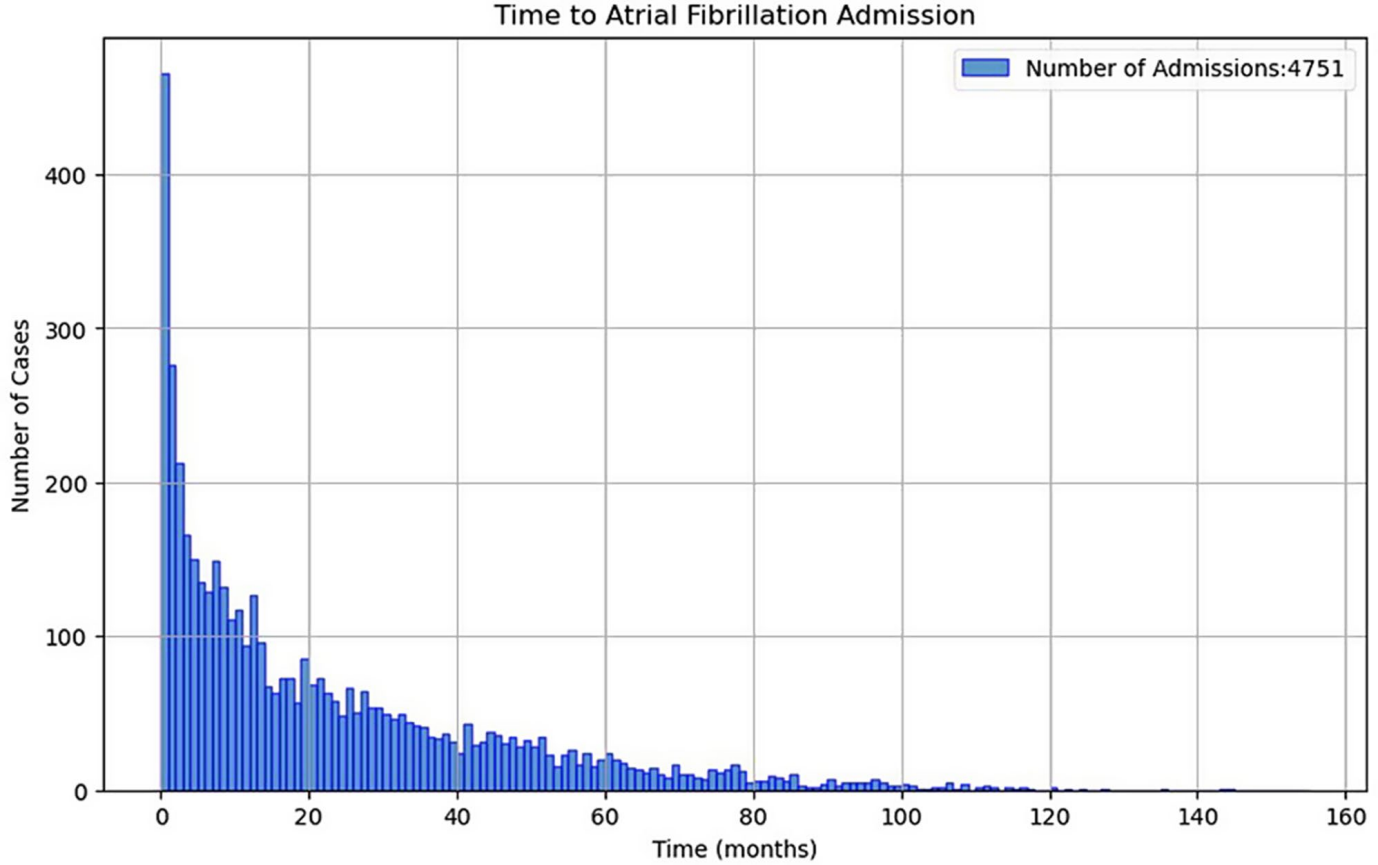
Histogram of incident admissions to the hospital with a primary diagnosis of atrial fibrillation over the follow-up period

A total of 219,667 patients were included in the dataset of whom 32,898 (15%) had a known prior history of AF. Of the remaining 186,769 patients, 4,751 (2.5%) had a hospital admission for primary diagnosis of AF, over a median follow-up of 35 months. Figure 2 shows a histogram of the time to first AF admission after the index Echo test.

Table 1 shows the baseline characteristics of patients included in this analysis, stratified by AF status. As can be noted, patients with AF were significantly older, were more likely to be men and white, and had significantly more cardiac and non-cardiac comorbidities compared to patients without incident AF admission during follow-up.

**Fig. 3:**
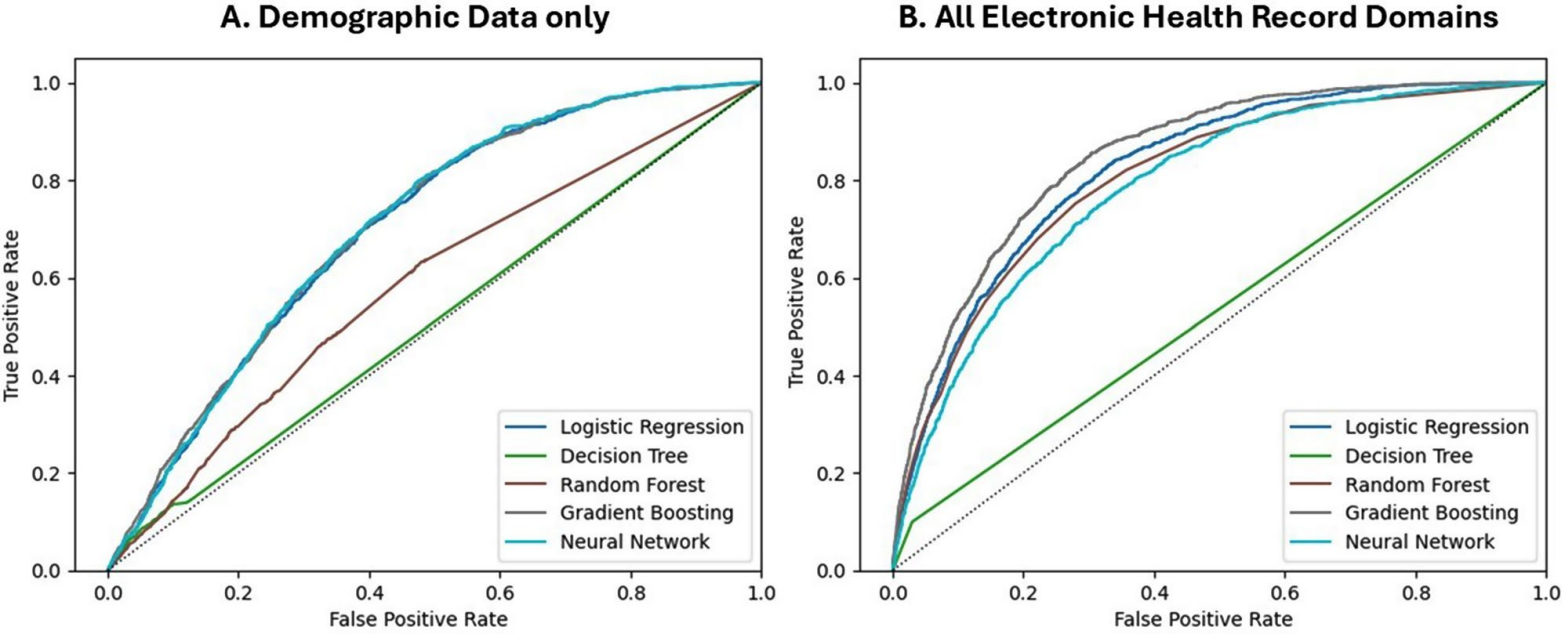
Receiver-operator curves for the prediction of incident AF hospital admission in AF-naïve patients, using demographic data only (panel **A**) versus using all structured electronic health record domains (panel **B**)

**Fig. 4:**
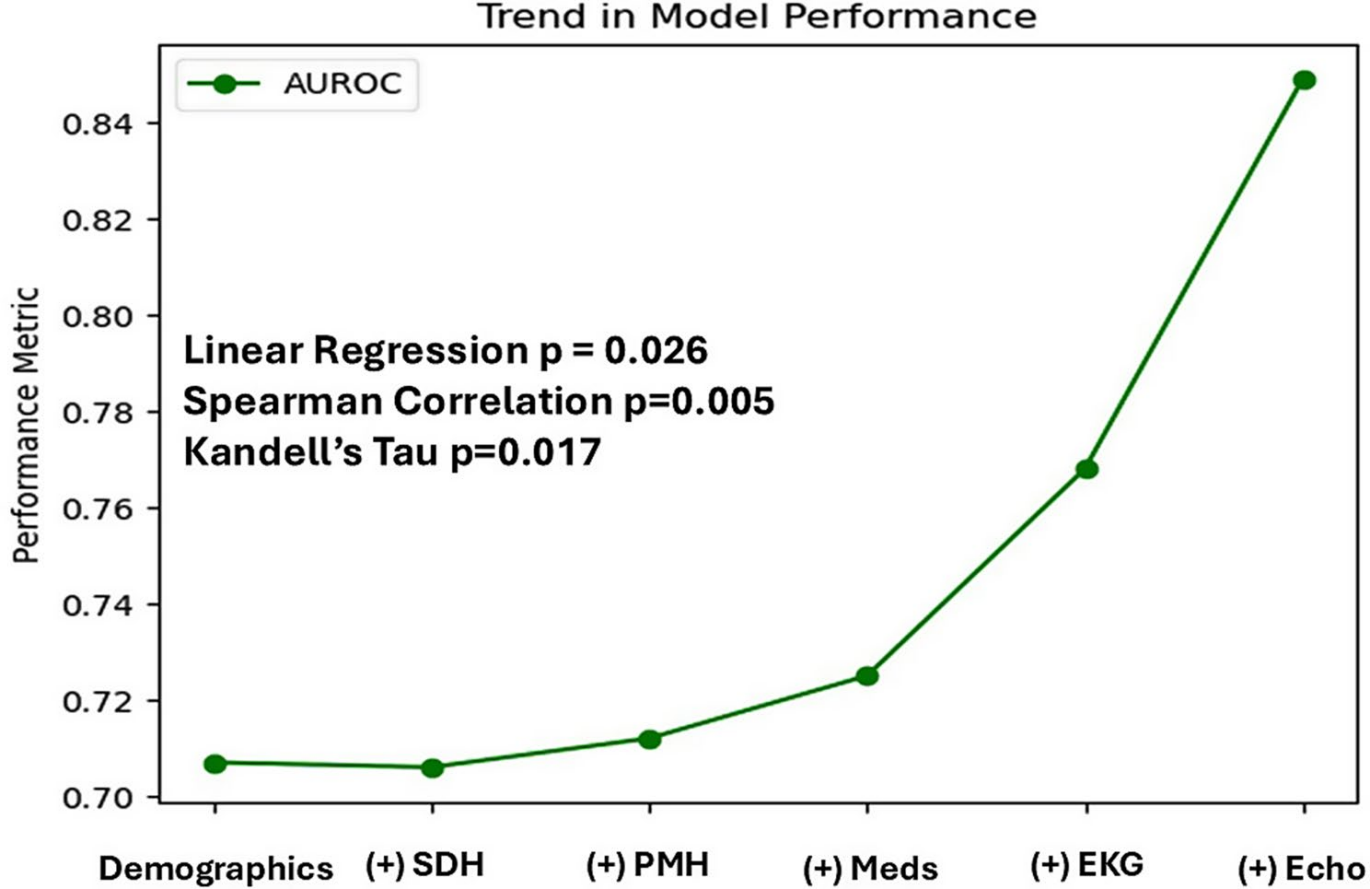
Changes in the area under the receiver-operator curve of the gradient boosting classifier with the inclusion of incremental domains of structured data from the electronic health records

Table 2 details the performance of ML models with incremental introduction of domains from the structured EHR data. Using the AUROC metric, the best performing ML models was gradient boosting, whereby the AUROC increased from 0.71 to 0.85 (Kendall Tau’s *p* = 0.017), with stepwise inclusion of more structured domains of EHR data (Figs. 3, 4). The performance of the best model, gradient boosting, at the optimal Youden threshold, with the use of all EHR domains compared to using only demographic data, resulted in reclassification of 11,877 out of 37,118 cases in the test cohort, or a reclassification rate of 32% with the use of all EHR domains, improving the model accuracy from 51% to 73%.

**Table 2:** Performance of the different machine learning algorithms in predicting incident admission to the hospital with atrial fibrillation by domain of structured electronic health record data calculated to maximize youden index

Model	Accuracy	AUROC	Sensitivity	Specificity	PPV	NPV	F1 Score
**Demographic Domain**							
Logistic Regression	0.500	0.702	0.824	0.492	0.042	0.990	0.080
Decision Tree	0.884	0.509	0.129	0.904	0.035	0.975	0.055
Random Forest	0.538	0.580	0.604	0.537	0.034	0.980	0.064
Gradient Boosting	0.511	0.707	0.817	0.503	0.042	0.990	0.081
Neural Network	0.509	0.704	0.813	0.501	0.042	0.990	0.080
**Social Determinants of Health**							
Logistic Regression	0.549	0.701	0.769	0.543	0.043	0.989	0.082
Decision Tree	0.943	0.506	0.045	0.967	0.036	0.974	0.040
Random Forest	0.361	0.625	0.832	0.349	0.033	0.987	0.064
Gradient Boosting	0.550	0.706	0.773	0.544	0.044	0.989	0.083
Neural Network	0.596	0.695	0.705	0.593	0.045	0.987	0.084
**Past Medical History**							
Logistic Regression	0.582	0.705	0.744	0.577	0.045	0.988	0.085
Decision Tree	0.941	0.505	0.046	0.965	0.034	0.974	0.039
Random Forest	0.573	0.654	0.665	0.571	0.040	0.984	0.076
Gradient Boosting	0.547	0.712	0.777	0.541	0.044	0.989	0.083
Neural Network	0.535	0.661	0.719	0.530	0.040	0.986	0.075
**Medications**							
Logistic Regression	0.574	0.720	0.769	0.569	0.046	0.989	0.087
Decision Tree	0.944	0.516	0.064	0.968	0.050	0.975	0.056
Random Forest	0.575	0.663	0.670	0.572	0.041	0.985	0.076
Gradient Boosting	0.536	0.725	0.801	0.528	0.044	0.990	0.083
Neural Network	0.540	0.671	0.734	0.535	0.041	0.987	0.077
**Electrocardiographic Data**							
Logistic Regression	0.634	0.746	0.734	0.632	0.051	0.989	0.095
Decision Tree	0.942	0.513	0.061	0.966	0.045	0.974	0.052
Random Forest	0.596	0.718	0.736	0.592	0.046	0.988	0.087
Gradient Boosting	0.637	0.768	0.763	0.634	0.053	0.990	0.100
Neural Network	0.568	0.699	0.739	0.563	0.044	0.988	0.083
**Echocardiogram Data**							
Logistic Regression	0.665	0.824	0.843	0.660	0.063	0.994	0.117
Decision Tree	0.947	0.535	0.101	0.970	0.082	0.976	0.090
Random Forest	0.721	0.804	0.751	0.720	0.067	0.991	0.124
Gradient Boosting	0.734	0.849	0.821	0.732	0.076	0.993	0.139
Neural Network	0.738	0.770	0.676	0.740	0.065	0.988	0.119

In addition, we investigated the performance of the model using only the EKG and Echo domains at the exclusion of all other EHR domains. As shown in Supplemental Table S1 and Supplemental Figure SF1, the model’s performance with only these 2 domains was equivalent to that of using all EHR domains, which has important implications to computational burden.

Despite the good performance of the prediction models when all available demographic and clinical information were incorporated, the F1 scores remained poor at about 14% for the gradient boosting classifier, indicating poor precision, owing primarily to the very low incidence of clinical events. To exclude model overfitting, we applied the gradient boosting test to the training set using all EHR domains and documented comparable accuracy (0.713) and AUROC (0.857) as when we used the testing set (Accuracy = 0.734 and AUROC = 0.849).

In addition, modeling the outcome as a survival analysis using incremental EHR domains was performed using Random Survival Forest. As shown in Supplemental Table S2, time-dependent models did not outperform other classifiers.

Lastly, we externally validated our prediction models using a cohort of 58,687 patients with no prior history of AF, who had an AF admission event rate of 5.8% over a median follow-up time of 58 months. Given missing variables and longer median follow-up time in the external validation dataset, we reran our model on both the derivation and external validation datasets, after excluding the 5 missing variables and by setting a fixed time horizon ( = 35 months). The resultant AUROC was 0.758 in the derivation dataset and 0.700 in the external validation dataset. At the Youden threshold, the external validation yielded an accuracy of 0.804 and an F1 score of 0.243.

## Discussion

Our data demonstrate that (1) there is incremental gain in AF prediction from adding more EHR domains to ML models but that most of the gain is achieved by using structured EKG and Echo data; (2) although ML models exhibited good discrimination in predicting AF from structured domains of the EHR, the precision is poor due to the low incidence of AF hospitalization events in our dataset. Predicting AF using only structured data from the EHR is therefore limited and may possibly be enhanced by including all AF events, not only ones leading to hospital admissions, as well as by incorporating non-structured EHR data, mainly digitized EKG tracings as well as images and/or video loops from Echo tests and other cardiac imaging modalities. This will have to be tested in future studies and remains speculative for the present time.

Predicting the incidence of AF using artificial intelligence has previously been attempted using digitized EKG tracings during normal sinus rhythm [14] and with markers of diastolic dysfunction on Echo [15] with good results. In a study by Atia et al. [14], predicting the presence of AF from normal rhythm tracings achieved an AUROC of 0.87 with an F1 score of 39.2%. To our knowledge however, applying ML algorithms to structured EHR data to predict incident AF hospital admissions and analyzing the contribution of various EHR domains has not been previously done. In addition, predicting AF admissions to the hospital selects for a sicker patient population, and to our knowledge, has not been previously undertaken.

Predicting incident AF before its actual occurrence has important clinical implications, given that AF is very common, can elude diagnosis over a long period of time and is often the cause of stroke or other serious thromboembolic events before an actual arrhythmia is diagnosed. The recently published ARCADIA trial [16, 17] randomized 1,015 patients with embolic stroke of unknown source to oral anticoagulation versus aspirin, with the goal of demonstrating that oral anticoagulation is superior to aspirin in preventing recurring stroke. Although patients in ARCADIA were enriched for the risk of developing AF by the presence of atrial cardiopathy, the trial failed to demonstrate the superiority of oral anticoagulation over aspirin in reducing the incidence of stroke, likely due to the selection criteria for AF risk. The premise of our study was therefore to examine the contribution of structured EHR domains to predicting AF.

The present study has limitations. First the data is extracted from a single center with no external validation, so our findings may not be generalizable to other patient populations. Still, our healthcare system comprises more than 40 hospitals across a wide geography spanning the commonwealth of Pennsylvania and beyond, therefore our study cohort likely has good representation of the general population. Second, although we excluded patients with prior known history of AF, we cannot be sure whether some of our patients may have had subclinical arrhythmia. Lastly, our clinical outcome for this study was hospital admission for AF and not any occurrence of AF, as this endpoint could be easily ascertained. This, however, has limited our event rate and has significantly impacted our average precision over the recall range. Future efforts from our group will focus on including any AF events, which may improve our model performance.

## Conclusions

In conclusion, our ML models exhibit good discrimination in predicting AF admissions from structured domains of the EHR, with the EKG and Echo domains contributing the most to model predictability. Still, the precision is poor due to the low incidence of clinical events.

## Electronic supplementary material

Below is the link to the electronic supplementary material.

Supplementary Material 1

## Data Availability

Data used in this study is part of the electronic health records of the University of Pittsburgh Medical Center and is stored within the institutional data warehouse. Deidentified data could be made available to other researchers upon reasonable request.

## Abbreviations

AF: Atrial fibrillation
AUROC: Area under the receiver operating characteristic
HER: Electronic health record
EKG: Electrocardiogram
ML: Machine learning
NPV: Negative predictive value
PPV: Positive predictive value
