# Supplementary material for: Prediction of atrial fibrillation admissions in arrhythmia naïve patients from structured electronic health record data
Source: BMC Med Inform Decis Mak. 2025 Sep 29;25:348. doi: 10.1186/s12911-025-03199-x (PMC12482350; doi:10.1186/s12911-025-03199-x)
Supplement: Supplementary file 1 — Supplementary Material 1 [file 12911_2025_3199_MOESM1_ESM.docx]

**Supplemental Tables and Figures**

**Supplemental Table S1. Model performance using only EKG and Echo domains**

| **Model** | **Accuracy** | **AUROC** | **Sensitivity** | **Specificity** | **PPV** | **NPV** | **F1 Score** |
| --- | --- | --- | --- | --- | --- | --- | --- |
| **Logistic Regression** | 0.726 | 0.789 | 0.718 | 0.727 | 0.066 | 0.990 | 0.121 |
| **Decision Tree** | 0.946 | 0.534 | 0.099 | 0.969 | 0.080 | 0.976 | 0.089 |
| **Random Forest** | 0.731 | 0.791 | 0.719 | 0.731 | 0.067 | 0.990 | 0.123 |
| **Gradient Boosting** | 0.737 | 0.829 | 0.777 | 0.735 | 0.073 | 0.992 | 0.134 |
| **Neural Network** | 0.766 | 0.817 | 0.722 | 0.767 | 0.077 | 0.990 | 0.140 |

**Supplemental Table S2. Performance of Random Survival Forest model in predicting time to admission to the hospital with atrial fibrillation by domain of structured electronic health record data calculated at time horizons**

| **Time Horizon** | **Accuracy** | **AUROC** | **Sensitivity** | **Specificity** | **PPV** | **NPV** | **F1 Score** |
| --- | --- | --- | --- | --- | --- | --- | --- |
| **DEMOGRAPHIC DOMAIN** | | | | | | | |
| **12 months** | 0.752 | 0.719 | 0.030 | 0.760 | 0.001 | 0.985 | 0.003 |
| **24 months** | 0.600 | 0.715 | 0.091 | 0.608 | 0.004 | 0.975 | 0.007 |
| **36 months** | 0.469 | 0.707 | 0.203 | 0.474 | 0.008 | 0.967 | 0.015 |
| **+ SOCIAL DETERMINANTS OF HEALTH** | | | | | | | |
| **12 months** | 0.769 | 0.720 | 0.027 | 0.778 | 0.001 | 0.985 | 0.003 |
| **24 months** | 0.604 | 0.718 | 0.092 | 0.613 | 0.004 | 0.976 | 0.008 |
| **36 months** | 0.472 | 0.712 | 0.207 | 0.478 | 0.008 | 0.967 | 0.015 |
| **+ PAST MEDICAL HISTORY** | | | | | | | |
| **12 months** | 0.836 | 0.727 | 0.021 | 0.846 | 0.002 | 0.986 | 0.003 |
| **24 months** | 0.625 | 0.722 | 0.070 | 0.634 | 0.003 | 0.976 | 0.006 |
| **36 months** | 0.482 | 0.713 | 0.187 | 0.488 | 0.007 | 0.967 | 0.014 |
| **+ MEDICATIONS** | | | | | | | |
| **12 months** | 0.798 | 0.744 | 0.016 | 0.807 | 0.001 | 0.986 | 0.002 |
| **24 months** | 0.621 | 0.737 | 0.060 | 0.630 | 0.003 | 0.975 | 0.005 |
| **36 months** | 0.483 | 0.728 | 0.178 | 0.489 | 0.007 | 0.967 | 0.014 |
| **+ ELECTROCARDIOGRAPHIC DATA** | | | | | | | |
| **12 months** | 0.791 | 0.789 | 0.011 | 0.800 | 0.001 | 0.985 | 0.001 |
| **24 months** | 0.588 | 0.778 | 0.079 | 0.596 | 0.003 | 0.975 | 0.006 |
| **36 months** | 0.449 | 0.768 | 0.180 | 0.454 | 0.007 | 0.964 | 0.013 |
| **+ ECHOCARDIOGRAM DATA** | | | | | | | |
| **12 months** | 0.728 | 0.812 | 0.014 | 0.736 | 0.001 | 0.984 | 0.001 |
| **24 months** | 0.546 | 0.816 | 0.055 | 0.555 | 0.002 | 0.972 | 0.004 |
| **36 months** | 0.385 | 0.817 | 0.129 | 0.390 | 0.004 | 0.956 | 0.008 |

**Supplemental Figure SF1.**

**Supplemental Figure F1.** ROC curves of the model performance using only the EKG and Echo domains of the electronic health records.

**
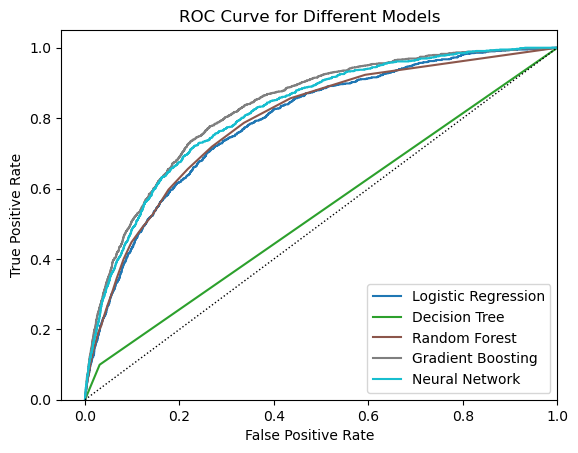
**
